# Secret of the Masters: Young Chess Players Show Advanced Visual Perspective Taking

**DOI:** 10.3389/fpsyg.2019.02407

**Published:** 2019-10-24

**Authors:** Qiyang Gao, Wei Chen, Zhenlin Wang, Dan Lin

**Affiliations:** ^1^Center for Brain, Mind and Education, Shaoxing University, Shaoxing, China; ^2^Department of Psychology, The Education University of Hong Kong, Tai Po, Hong Kong

**Keywords:** chess, visual perspective taking, executive function, egocentric bias, altercentric bias

## Abstract

Playing chess requires perspective taking in order to consistently infer the opponent’s next moves. The present study examined whether long-term chess players are more advanced in visual perspective taking tasks than their counterparts without chess training during laboratory visual perspective taking tasks. Visual perspective taking performance was assessed among 11- to 12-year-old experienced chess players (*n* = 15) and their counterparts without chess training (*n* = 15) using a dot perspective task. Participants judged their own and the avatar’s visual perspective that were either consistent with each other or not. The results indicated that the chess players out-performed the non-chess players (Experiment 1), yet this advantage disappeared when the task required less executive functioning (Experiment 2). Additionally, unlike the non-chess players whose performance improved in Experiment 2 when the executive function (EF) demand was reduced, the chess players did not show better perspective taking under such condition. These findings suggested that long-term chess experience might be associated with children’s more efficient perspective taking of other people’s viewpoints without exhausting their cognitive resources.

## Introduction

One key benefit of chess training is the constant contemplation of the opponent’s next moves by taking on the opponent’s perspective. Perspective taking ability, sometimes referred to as theory of mind (ToM), is the ability to infer other people’s mental states in order to explain and predict behavior (Galinsky et al., 2005; Stiller and Dunbar, 2007). Perspective taking emerges early in life and develops throughout childhood and adolescence (Woodward, 1998; Meltzoff, 2005; Hamlin et al., 2007; Wang et al., 2016). Even adults show considerable individual differences in perspective taking abilities (Stiller and Dunbar, 2007; Cabello et al., 2016). Such individual difference influences how we successfully navigate our social environment. For instance, previous studies had demonstrated the link between children’s ToM and communicative competence (Slaughter et al., 2013), prosocial behavior (Imuta et al., 2016), and bullying (Baxevani and Metallidou, 2015; Álvarez-García et al., 2015). However, we know relatively little about what drives the individual differences in people’s perspective taking ability.

Several studies indicated that the quality and quantity of social input contribute to individual differences in ToM development in children. For instance, sibling studies suggested that having a child-aged sibling is beneficial to ToM development (Hughes and Devine, 2015). Deaf children from hearing families were found to demonstrate ToM delay, whereas deaf children growing up with deaf parents were not, indicating the importance of functional communicative input for ToM development (Peterson and Siegal, 2000). Training studies had shown that exposure to certain narrative practices was associated with improved ToM performance (Hofmann et al., 2016). Examples include engaging children in conversations that are rich in discussion of mental states and reading literary fiction, which help children become aware of alternative perspectives (e.g., Kidd and Castano, 2013). In the current study, we were interested in the relationship between chess experience and children’s perspective taking ability.

### Perspective Taking, Executive Function, and Chess

Chess research from a cognitive and developmental psychology perspective has traditionally focused on basic cognitive processes such as memory, representation, and problem solving strategy (Chase and Simon, 1973; Chi, 1978; Saariluoma, 2001). Nevertheless, chess research deserves more attention since chess playing provides a model task environment that is ideal for studying more complicated processes (Charness, 1992). In terms of perspective taking, researchers often think of chess as an iterative process of putting ourselves in the opponents’ “mind” (Camerer et al., 2005). Just like other game theory protocols (e.g., the Prisoners Dilemma, the Dictator Game, and the Ultimatum games) that require inferences of another’s mental states (de Weerd et al., 2017; Ghosh et al., 2017), playing chess involves reasoning iteratively about the opponent’s potential intentional choices. Neurologically, playing chess and performing a perspective taking task involve the same brain areas. For example, Powell et al. (2017) observed that novice chess players activated cortical structures that corresponded to ToM-related regions while playing. Specifically, when chess players estimated the possible mental reasoning of the opponent, their right temporoparietal junction (TPJ) was activated, which is considered to have a significant role in differentiating self from others (Lawrence et al., 2006). Other studies noted the role of fusiform gyrus in chess playing (Bukach et al., 2006), which is also activated in the Level-1 visual perspective taking task (Schurz et al., 2015).

Chess experience is associated with not only perspective taking ability, but also executive function (EF) (Unterrainer et al., 2006). Those with more advanced EF process information faster, inhibit irrelevant information more effectively, and correct their own mistakes more swiftly. In healthy individuals, playing chess was associated with increased prefrontal cortex activation, an area related to EF (Nichelli et al., 1994; Atherton et al., 2003). In clinical samples, practicing chess for 4 weeks improved EF in patients with schizophrenia and cocaine dependency (Demily et al., 2009; Goncalves et al., 2014). The questions remain on just how exactly chess experience affects perspective taking, and what role EF plays in this process.

### Egocentric Bias and Altercentric Bias

Taking another person’s perspective requires cognitive resources and conceptual competences. Knowledge possessors tend to assume that others share the same knowledge base (Goldman, 2006). This is referred to as an egocentric bias—a tendency to overestimate how similar other people’s experiences are to one’s own (Frith and De Vignemont, 2005; Goldman and Sebanz, 2005), sometimes referred to as *the curse of knowledge* (Birch and Bloom, 2007). Egocentric interferences are most salient during complicated tasks or those that require certainty judgments regarding other’s behaviors or thoughts. For example, both adults and older children show a tendency to overestimate the intersection of their own attitudes and emotions with others’ estimation of such (Ross et al., 1977; Krueger and Clement, 1994). Specifically, people have the tendency to believe that others have more access to their internal states than others actually do (Gilovich et al., 1998, 2000). In other words, we use our own knowledge as a roadmap to understand other people’s knowledge (Keysar et al., 2000, 2003). Furthermore, observers tend to make more errors when interpreting instructions according to another person’s perspective than according to an arbitrary rule (Apperly et al., 2010).

Egocentric bias arises from the pervasive failures in making appropriate adjustments from an anchor of our own first-person phenomenological experiences (Macrae et al., 2016). Perspective taking aims to overcome such bias by trying to perceive a situation from another person’s point of view (Eyal et al., 2018). Existing research had found one simple solution for diminishing egocentric bias, that is, to put oneself in another person’s shoes and imagine another’s thoughts and feelings from their perspective (Miles et al., 2014; Golubickis et al., 2016; Macrae et al., 2016; Eyal et al., 2018).

Different from egocentric bias, altercentric bias in perspective taking arises due to the influence of others’ mental states on our judgments of own perspective (Samson et al., 2010). For instance, judgments of one’s own visual experience could be affected by the viewpoint of another (i.e., an avatar) (Samson et al., 2010). Unlike the slow and effortful process of overcoming egocentric biases, simple visual perspective taking is rapid and effortless (Samson et al., 2010, but see Ferguson et al., 2017 for a different view). Altercentric bias is a cognitively efficient competence for engaging in simple perspective taking among both adults and children (Surtees and Apperly, 2012).

### The Current Study

The egocentric bias and altercentric bias reflect the two aspects of interference during perspective taking in chess. It is reasonable to hypothesize that children with chess playing experience would demonstrate more automated perspective taking ability, as well as diminished egocentric and enhanced altercentric biases. A competing hypothesis would be that experienced chess players might display better perspective taking abilities simply through more effective inhibition of egocentric biases.

In two experiments, long-term chess players and their counterparts without chess experience judged their own and the avatar’s perspectives. The two experiments differed in their demands on the participants’ EF. Switching perspectives is cognitively effortful, while assessing another’s perspective is less so (Qureshi et al., 2010; Bradford et al., 2015; Ferguson et al., 2017). The design of Experiment 1 required participants to switch between self-perspective and other perspective within the same blocks. In Experiment 2, however, the participants was not required to switch perspectives within each block but still needed to do so between blocks. Manipulating the perspective switching from within blocks in Experiment 1 to between blocks in Experiment 2 enables EF demand reduction. If the benefit of chess experience in children’s perspective taking rested on more automated process, the participants’ performance pattern should be similar in Experiment 1 and in Experiment 2, and the chess players should outperform their counterparts in both experiments. However, if the competing hypothesis was true and the benefit was due to more efficient switching and inhibition, reducing the EF demands in Experiment 2 should eliminate the chess players’ advantage over the non-chess players. Specifically, both groups would show a larger egocentric intrusion than altercentric intrusion in Experiment 2.

We adapted Samson et al.’s (2010) “avatar” visual perspective taking task. The task was widely used to test Level-1 perspective taking performance across ages (Qureshi et al., 2010; Samson et al., 2010; Surtees and Apperly, 2012; Nielsen et al., 2015; Furlanetto et al., 2016; Ferguson et al., 2017). Participants were told to judge either the number of red disk that they could see on the walls (Self-perspective condition) or the number that could be seen by an avatar standing in the room (Other-perspective condition). For consistent trials, both the participant and avatar could see the same number of disk. For inconsistent trials, the participant and avatar each saw a different number of disk. Slower response times and more errors in the inconsistent condition compared to the consistent condition when judging the avatar’s perspective would indicate egocentric intrusions. Conversely, slower response times and more errors in the inconsistent condition compared to the consistent condition when judging self-perspective would indicate altercentric intrusions.

## Experiment 1

### Materials and Methods

#### Participants

Thirty children aged between 11- and 12-years (*M*age = 11.64 years) were recruited from a primary school for the experiment. All participants were right-handed and had normal or corrected-to-normal vision. Deliberate practice was shown to be a very important factor in determining individual player’s chess skill (Ericsson et al., 1993; Campitelli and Gobet, 2011), in addition to cognitive abilities such as general intelligence and EFs (Burgoyne et al., 2016). In the current study, the 15 chess players were selected based on the fact that these children had long-term deliberate and intensive chess training. As members of the school chess team, the majority of the chess players started formal chess training when they were 6-years-old through self-expressed interest and teacher recommendations. They were trained for approximately 2 h a day for each and every school day in face to face sessions in the last four to 5 years, which means each player had accumulated more than 1,000 h of training. A comparable sample of 15 children from the same school was recruited through teacher nomination. We asked the classroom teachers to nominate students who were comparable with the chess players on academic performances but not in the school chess team. The two groups were matched on age, gender, the latest grades on the subjects of mathematics and Chinese, and teacher’s ratings of their academic performances on mathematics and Chinese on a 5-point Likert scale (1 = top 50%; 2 = top 40%; 3 = top 30%; 4 = top 20%; and 5 = top 10%). Table 1 presented the descriptive statistics of the participants’ details. A series of *t*-tests confirmed that the two groups did not differ on age, *t*(28) = 0.454, *p* = 0.634; mathematics grade, *t*(28) = −0.964, *p* = 0.344; Chinese grade, *t*(28) = 0.459, *p* = 0.650; teacher rating for mathematics, *t*(28) = −0.418, *p* = 0.679; or teacher rating for Chinese *t*(28) = 0.386, *p* = 0.702.

**TABLE 1:** Participant details.

	**Sex**		**Age**		**Math performance**				**Chinese performance**			
						
					**Grades**		**Teacher rating**		**Grades**		**Teacher rating**	
								
	***n***	**B/G**	***M***	***SD***	***M***	***SD***	***M***	***SD***	***M***	***SD***	***M***	***SD***
CG	15	7/8	11.62	1.45	93.00	4.03	4.73	0.46	92.00	3.29	4.73	0.45
NCG	15	7/8	11.66	1.38	94.26	3.10	4.80	0.41	91.47	3.06	4.66	0.48

*CG, chess group; NCG, non-chess group; B, boys; G, girls.*

#### Stimuli and Procedure

The study was approved by the Research Ethics Committee of Shaoxing University and the principals of the participating schools. This study adopted a single-blind design. The experimenter was aware of the purpose of experiment and the participants’ group membership. The participants were told that they would complete a computer task, without information about the purpose or the design of the study. The experimenter explained to the participants the voluntary nature of their participation. Written informed consents were obtained from the parents of the participating children.

The participants were given a picture showing the lateral view of a room, with the left, right, and back walls visible. A male avatar created by the three-dimensional animation software *Poser 6* was positioned in the center of the room, always facing either the left or the right wall. A certain number of red dots (0, 1, 2, or 3) were randomly displayed on the left or right walls (or both). For 50% of the trials, the number of red dots perceived by the participants and the avatar were the same; for the rest of the trials, the number seen by the participants and the avatar were different. The standing position of the avatar and dot display remained constant in both conditions.

The experiment was presented with E-prime 2.0 software. The participants were first given instructions on the procedures and how to respond to the stimuli. Each trial contained four steps. First, a fixation cross (+) was presented for 750 ms followed by a 500 ms blank screen. Secondly, a Chinese word 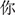 (You) or 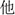 (He) was presented for 750 ms as a cue for which perspective should be taken next. When the word 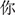 (You) was presented, the participants were required to make the judgment from their own perspective (Self condition); when the word 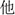 (He) was presented, the participants were required to do so from the avatar’s perspective (Other condition). In the third step, after another 500 ms interval, a digit ranging from 0 to 3 was presented for 750 ms, specifying the number of red dots for the participants to verify. Finally, the picture of the room was presented until the participants pressed one of two keys to judge whether the picture matched (“Yes” response) or mismatched (“No” response) the given content from the given perspective. The participants were instructed to press “*F*” on the keyboard when the picture matched and “*J*” when the picture mismatched. If the participants did not respond after 2,000 ms, the next trial automatically appeared. The participants were encouraged to respond accurately and their response time and accuracy were analyzed.

### Design and Analysis

A three-factor mixed-design (Perspective: self, avatar; Consistency: consistent, inconsistent; and Group: chess, non-chess) experiment was implemented. Overall, the trials were divided into 4 blocks of 52 test trials each (48 test trials and 4 filler trials), with 16 practice trials preceding the actual test. There were 48 matching trials from self-perspective and another 48 matching trials from the avatar’s perspective, each including 24 consistent and 24 inconsistent trials. An equal number of mismatching trials including consistent and inconsistent ones were also included. The 16 fillers trials showed no red dots in the picture, hence the correct response was “0.” Within each block, trial order was pseudo-randomized so that there were no more than three consecutive trials of the same type. Self and other trials were equally preceded by the same given perspective (no shift in perspective) and by a different perspective (shift in perspective).

Notably, for the consistent and inconsistent trials in the matching condition (i.e., “Yes” responses), the given digit always corresponded to the number of red dots from the given perspective (either self or other condition). In the inconsistent mismatching (i.e., “No” responses) trials, however, the given digit always corresponded to the number of red dots appearing from the irrelevant perspective (i.e., the number of red dots seen by the avatar when participants were asked to judge from their own perspective, or that seen from their own perspective when asked to judge from the avatar’s perspective). In the consistent mismatching trials (i.e., “No” responses), the given digit did not correspond to either the participant’s or the avatar’s perspective. The purpose of including the mismatching trials was to balance the responses. It is the matching trials that are meaningful in understanding the participants’ egocentric bias and altercentric bias. Following Samson et al. (2010), Qureshi et al. (2010), and Ferguson et al. (2017)’s procedure, only data from the matching trials were analyzed.

### Results

We conducted a 2 (Perspective) × 2 (Consistency) × 2 (Group) mixed repeated measure analysis of variance (ANOVA) with Perspective (Self vs. Other) and Consistency (Consistent vs. Inconsistent) as within-subject factors and Group (Chess vs. Non-chess) as a between-subject factor. Reaction times (RTs) and error rates were considered independently for in-depth analysis. Table 2 displayed the mean RTs and error percentages in each condition of Experiment 1.

**TABLE 2:** Experiment 1 descriptive statistics.

**Perspective**	**Consistency**	**Reaction time analysis**		**Error Analysis**	
			
		**Chess**	**Non-chess**	**Chess**	**Non-chess**
Self	Consistent	742 (122.67)	805 (179.98)	1.87% (0.003)	5.27% (0.006)
	Inconsistent	819 (168.47)	860 (178.52)	4.47% (0.005)	13.73% (0.011)
Other	Consistent	763 (132.12)	794 (216.95)	3.20% (0.004)	10.27% (0.007)
	Inconsistent	844 (166.05)	939 (208.96)	8.60% (0.060)	19.93% (0.013)

*Means (standard errors).*

#### RT Analysis

The RT analysis only included trials that the participants responded correctly. The main effect of Perspective was significant, *F*(1, 28) = 4.93, *MSE* = 24196.80, *p* = 0.035, ${\text{η}}_{p}^{2}$ = 0.15. The participants reacted significantly faster when judging from self-perspective (*M* = 780 ms) than from other-perspective (*M* = 803 ms). The main effect of Consistency was also significant, *F*(1, 28) = 43.46, *MSE* = 239592.03, *p* < 0.001, ${\text{η}}_{p}^{2}$ = 0.61. The participants were significantly slower in the inconsistent condition (*M* = 831 ms) than in the consistent condition (*M* = 753 ms). However, the main effect of Group was not significant, *F*(1, 28) = 0.90, *MSE* = 98498.70, *p* = 0.350, ${\text{η}}_{p}^{2}$ = 0.03 There was a significant interaction between Perspective and Consistency, *F*(1, 28) = 43.47, *MSE* = 239592.03, *p* = 0.000. Paired *t*-tests revealed a significant Consistency effect when the participants judged the avatar’s perspective, *t*(29) = 6.199, *p* = 0.000, with a 112 ms advantage in the consistent condition. There was also a significant but numerically smaller Consistency effect when participants judged their own perspective in the consistent condition, *t*(29) = 4.596, *p* = 0.000, with a 66 ms advantage. There were no other significant interactions (all *Fs* ≤ 0.62, *ps* ≥ 0.441, ${\text{η}}_{p}^{2}$s ≥ 0.021).

Furthermore, the three-way interaction was significant, *F*(1, 28) = 6.38, *MSE* = 14083.33, *p* = 0.017, ${\text{η}}_{p}^{2}$ = 0.19. The three-way interaction was followed up with a 2 (Perspective) × 2 (Consistency) ANOVAs for both the chess and non-chess group. For the chess group, the main effect of Consistency was significant, *F*(1, 14) = 18.57, *MSE* = 93062.82, *p* = 0.001, ${\text{η}}_{p}^{2}$ = 0.57. The participants were slower in the inconsistent condition (*M* = 831 ms) than in the consistent condition (*M* = 752 ms). However, the main effect of Perspective was not significant, *F*(1, 14) = 3.71, *MSE* = 7912.02, *p* = 0.075, ${\text{η}}_{p}^{2}$ = 0.21, as was the Consistency × Perspective interaction, *F*(1,14) = 0.025, *MSE* = 33.75, *p* = 0.877, ${\text{η}}_{p}^{2}$ = 0.002. Regarding the non-chess group, the main effect of Consistency was significant, *F*(1, 14) = 24.93, *MSE* = 149900.02, *p* < 0.001, ${\text{η}}_{p}^{2}$ = 0.64. The participants were significantly slower in the inconsistent condition (*M* = 831 ms) than in the consistent condition (*M* = 753 ms). The main effect of Perspective was not significant, *F*(1, 14) = 2.24, *MSE* = 17170.42, *p* = 0.157, ${\text{η}}_{p}^{2}$ = 0.21. The Consistency × Perspective interaction was significant, *F*(1, 14) = 9.896, *MSE* = 30150.42, *p* = 0.007, ${\text{η}}_{p}^{2}$ = 0.414. Paired *t*-tests revealed a significant Consistency effect when the participants judged the avatar’s perspective, *t*(14) = 5.014, *p* = 0.000, with a 144 ms advantage in the consistent condition. There was also a significant, albeit numerically smaller, Consistency effect when participants judged their own perspective, *t*(14) = 2.851, *p* = 0.013, with a 55 ms advantage in the consistent condition. Furthermore, only in the inconsistent condition was there a significant difference between self- and other-perspectives, *t*(14) = 3.589, *p* = 0.003, whereby RTs were 78 ms faster for self-perspective than for other-perspective. There was no significant difference between self-and other-perspective in the consistent condition, *t*(14) = 0.357, *p* = 0.726.

#### Error Analysis

A mixed ANOVA was conducted on error rates. A significant main effect of Group emerged, *F*(1, 28) = 15.707, *MSE* = 0.181, *p* < 0.001, ${\text{η}}_{p}^{2}$ = 0.359, indicating that the chess players made fewer errors (*M* = 4.5%) than the non-chess players (*M* = 12.3%). The main effect of Consistency was also significant, *F*(1, 28) = 22.644, *MSE* = 0.128, *p* < 0.001, ${\text{η}}_{p}^{2}$ = 0.447, with the participants making more errors when the two perspectives were inconsistent (*M* = 11.7% errors) than that when the two were consistent (*M* = 5.1% errors). The main effect of Perspective was significant, *F*(1, 28) = 14.241, *MSE* = 0.052, *p* = 0.001, ${\text{η}}_{p}^{2}$ = 0.337, with the participants making more errors when they judged from other-perspective (*M* = 10.5% errors) than from self-perspective (*M* = 6.3% errors). No other effects or interactions were significant.

### Discussion

The results suggested the existence of both egocentric and altercentric intrusions. When judging from the avatar’s perspective, the participants were affected by their own visual experience (egocentric intrusion effect); however, when judging from their own perspective, the participants were affected by the avatar’s perspective (altercentric intrusion effect). These results indicated that the participants were unable to inhibit an irrelevant perspective.

Differences in RTs between the chess group and non-chess group were particularly interesting. For the non-chess group, egocentric intrusions entailed greater interference than altercentric intrusions in the inconsistent trials, which was in line with prior findings in normal non-chess-playing adults, presumably (Samson et al., 2010). When the tasks were getting more difficult, non-chess players processed self-perspective faster than other perspective. The chess players, however, did not show this differentiation; they fended off egocentric intrusions and altercentric intrusions equally. It seems that children with long-term chess experience developed automatic tendencies to engage in other’s perspectives which became similar to the judgment of their own perspective.

The chess players made less errors than the non-chess players. Yet there were no interaction effects in the response accuracy, as that in the RTs. A closer scrutiny of the data showed that there were few errors for both groups, indicating a ceiling effect. The results were consistent with Samson et al.’s (2010) that showed egocentric intrusions caused larger interference than the altercentric intrusions did in RTs, and both types of intrusions interfered equally when taking into account of the participants’ accuracy, indicating that the difference between the two conditions rests on RTs. The current results were also consistent with Ferguson et al. (2017), which found a significant three-way Condition × Perspective × Consistency interaction in RTs, but not in response accuracy.

It is possible that the aforementioned results were influenced by the experimental paradigm. For instance, the participants were given a cue regarding the perspective they should take next before being presented with the stimulus. Within each block, both self-perspective and other-perspective trials were mixed and randomly presented. Such design demands higher level of EF. Taken into consideration that children with extensive chess experience were more likely to be efficient with shifting perspectives, their more advanced performance in this task was not surprising. An alternative possibility is that the group differences were the result of the chess players’ automated perspective taking abilities. This could be why the chess players were as quick and accurate at making explicit judgments about the avatar’s visual experience as that of their own.

In order to investigate these two competing possibilities, Experiment 2 implemented a modified paradigm, with the self-perspective and other-perspective trials being presented in separate blocks. Before each block, participants were cued as to which perspective they should take for the upcoming trials; thus, the alternative perspective did not need to be considered within a single block. Although this did not completely eliminate the EF demands since perspective switching between blocks was still present in Experiment 2, it did reduce the EF demands within the same blocks.

## Experiment 2

### Participants

The same participants from Experiment 1 participated in Experiment 2 two weeks later.

### Stimuli and Procedure

Experiment 2 presented self- and other-perspective trials in separate blocks. Prior to each block, the participants were told which perspective they should take during the subsequent block. Other than this change, the rest of the stimuli and procedure were the same as that in Experiment 1. The two self-perspective and two other-perspective blocks were presented alternatively, with half of the participants beginning with self-perspective and then other-perspective, and the other half doing the opposite.

### Results

**FIGURE 1:**
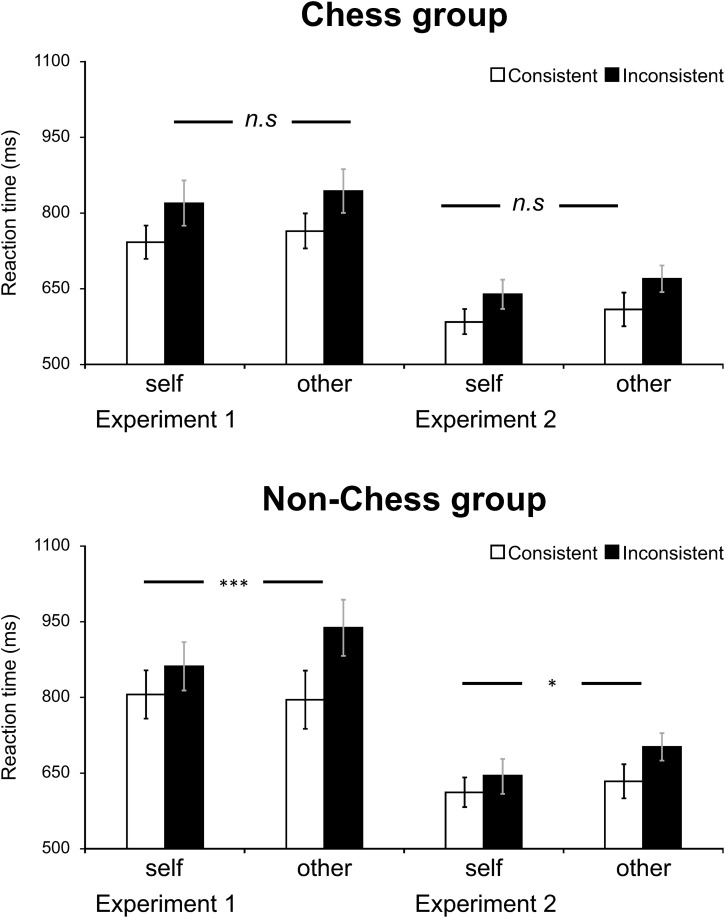
Mean response time for each experimental condition in Experiment 1 and Experiment 2. Symbols indicate significance level (^∗∗∗^*p* < 0.001; ^∗∗^*p* < 0.01; ^∗^*p* < 0.05; n.s = non significant).

Similar to Experiment 1, we performed a 2 (Consistency) × 2 (Perspective) × 2 (Group) mixed-design repeated measure ANOVA (see Table 3 for descriptive statistics). We also added Experiment (Experiment 1 vs. Experiment 2) as an additional within-subject factor to test the effect of EF demand in perspective shifting. An additional set of 2 (Consistency) × 2 (Perspective) repeated measures ANOVAs for the chess group and the non-chess group on RTs and error rates were added for the convenience of making sense of the results. Figure 1 displayed the mean RTs and errors rates in each experimental condition.

**TABLE 3:** Experiment 2 descriptive statistics.

**Perspective**	**Consistency**	**Reaction time analysis**		**Error Analysis**	
			
		**Chess**	**Non-chess**	**Chess**	**Non-chess**
Self	Consistent	584 (93.88)	619 (109.28)	3.50% (0.008)	3.80% (0.021)
	Inconsistent	638 (109.14)	650 (130.73)	6.30% (0.013)	8.70% (0.023)
Other	Consistent	608 (123.40)	642 (125.21)	4.50% (0.013)	4.40% (0.016)
	Inconsistent	670 (98.10)	706 (106.22)	10.20% (0.023)	9.00% (0.023)

*Means (standard errors).*

#### RT Analysis

As that in Experiment 1, incorrect responses were omitted in the RT analysis. The (Consistency) × 2 (Perspective) × 2 (Group) mixed-design repeated measure ANOVA revealed a significant main effect of Perspective, *F*(1, 28) = 7.689, *MSE* = 33835.21, *p* = 0.010, ${\text{η}}_{p}^{2}$ = 0.215. The participants reacted significantly faster when judging from self-perspective (*M* = 622 ms) than from other-perspective (*M* = 656 ms). The main effect of Consistency was significant, *F*(1, 28) = 60.248, *MSE* = 83371.41, *p* = 0.000, ${\text{η}}_{p}^{2}$ = 0.683. The participants were significantly slower in the inconsistent condition (*M* = 666 ms) than in the consistent condition (*M* = 613 ms). There was no effect of Group as a between-subjects factor, *F*(1, 28) = 0.590, *MSE* = 83371.41, *p* = 0.449, ${\text{η}}_{p}^{2}$ = 0.021, nor were there any significant interactions, *Fs* ≤ 3.047, *ps* ≥ 092, ${\text{η}}_{p}^{2}$s ≤ 0.098.

Pulling data from Experiments 1 and 2 together, separate ANOVAs for the chess group and the non-chess group were conducted with Experiment as a within-subject factor. There was a main effect of Experiment for the chess group, *F*(1, 28) = 13.83, *MSE* = 837672.30, *p* = 0.001, ${\text{η}}_{p}^{2}$ = 0.331. The participants were faster in Experiment 2 (*M* = 625 ms) than Experiment 1 (*M* = 792 ms). The main effect of Perspective was significant, *F*(1, 28) = 9.095, *MSE* = 19304.03, *p* = 0.005, ${\text{η}}_{p}^{2}$ = 0.245. The participants reacted significantly faster when judging from self-perspective (*M* = 696 ms) than from other-perspective (*M* = 721 ms). The main effect of Consistency was significant, *F*(1, 28) = 47.228, *MSE* = 140630.53, *p* = 0.000, ${\text{η}}_{p}^{2}$ = 0.628. The participants were significantly slower in the inconsistent condition (*M* = 743 ms) than in the consistent condition (*M* = 674 ms). No other effects or interactions emerged (all *Fs* ≤ 0.31, *ps* > 0.139, ${\text{η}}_{p}^{2}$s ≤ 0.001).

For the non-chess group, there was also a main effect of Experiment, *F*(1, 14) = 12.33, *MSE* = 1140555.01, *p* = 0.002, ${\text{η}}_{p}^{2}$ = 0.306. The participants were faster in Experiment 2 (*M* = 654 ms) than in Experiment 1 (*M* = 849 ms). The main effect of Perspective was significant, *F*(1, 28) = 5.600, *MSE* = 40223.41, *p* = 0.025, ${\text{η}}_{p}^{2}$ = 0.167. The participants reacted significantly faster when judging from self-perspective (*M* = 733 ms) than from other-perspective (*M* = 770 ms). The main effect of Consistency was significant, *F*(1, 28) = 41.490, *MSE* = 162582.41, *p* = 0.000, ${\text{η}}_{p}^{2}$ = 0.597. The participants were significantly slower in the inconsistent condition (*M* = 788 ms) than in the consistent condition (*M* = 714 ms). There was a significant interaction between Experiment and Consistency, *F*(1, 14) = 5.32, *MSE* = 20829.68, *p* = 0.029, ${\text{η}}_{p}^{2}$ = 0.160. Paired *t*-tests revealed a significant Consistency effect in Experiment 1, *t*(14) = 4.993, *p* < 0.001, with a 100 ms advantage in the consistent condition, and a significant but smaller Consistency effect in Experiment 2, *t*(14) = 4.285, *p* = 0.01, with a 47 ms advantage in the consistent condition.

#### Error Analysis

A mixed ANOVA with Perspective and Consistency as within-subjects factors, and Group as a between-subject factor revealed a significant main effect of Consistency, *F*(1,14) = 10.511, *MSE* = 0.028, *p* = 0.006, ${\text{η}}_{p}^{2}$ = 0.429, with the participants making more errors when the two perspectives were inconsistent (*M* = 8.6% errors) than when they were consistent (*M* = 4.0% errors). No other main effects or interactions were significant (all *Fs* < 1.233, *ps* > 0.276, ${\text{η}}_{p}^{2}$s < 0.042).

In assessing the combined data of Experiments 1 and 2, the chess group demonstrated no significant differences in accuracy across experiments, *F*(1, 28) = 1.142, *MSE* = 0.008, *p* = 0.294, ${\text{η}}_{p}^{2}$ = 0.039, nor did Experiment modulate any of the other variables (all *Fs* < 0.031, *ps* ≥ 0.862, ${\text{η}}_{p}^{2}$s < 0.001). The non-chess group demonstrated a main effect of Experiment, *F*(1,28) = 5.673, *MSE* = 0.102, *p* = 0.024, ${\text{η}}_{p}^{2}$ = 0.168, with the participants making more errors in Experiment 1 (*M* = 12.3% errors) than in Experiment 2 (*M* = 6.5% errors). However, Experiment type did not modulate any of the other variables (all *Fs* ≤ 3.869, *ps* ≥ 0.059, ${\text{η}}_{p}^{2}$s ≤ 0.121).

### Discussion

RT and error rate analyses demonstrated that egocentric and altercentric intrusions still remained in both groups. Although there was no perspective switching within each block of trials and the participants were explicitly instructed to take a specific perspective in advance, they still found it challenging to ignore the irrelevant perspective. Interestingly, when the perspective switching within blocks was eliminated from Experiment 2, the chess players’ advantage over the non-players on equally fending off both egocentric intrusions and altercentric intrusions reported in Experiment 1 disappeared. The two groups performed similarly and both showed faster RTs when judging from self perspective than from other perspective. However, the two groups still differ in other aspects of their performances. While the non-chess players demonstrated fewer intrusions in the non-switch condition of Experiment 2 than that in the switch condition of Experiment 1, in line with Ferguson et al. (2017) results, the presence of perspective switching did not influence the size of interference for the chess players.

## General Discussion

The current study explored the benefit of childhood chess experience on perspective taking abilities. Results indicated that although young chess players were affected by both egocentric intrusions and altercentric intrusions just like non-players, they were just as adept at taking another’s perspective as taking their own perspective, especially when the tasks required constant switching between perspectives. In a chess game, players need to constantly take the opponent’s perspective into consideration to adequately anticipate the next moves while planning their own moves and counter-moves from own perspective. In other words, considering the opponent’s perspective and own perspective simultaneously is a must in chess. Extensive training over the years helps young players perform perspective switching faster and more effectively. Taken together, the effect of chess training seems to be associated with children’s more efficient perspective taking of other people’s viewpoints without exhausting their cognitive resources.

The results contributed to the understanding of visual perspective taking mechanism. Firstly, our results replicated previous findings that visual perspective switching is effortful (Samson et al., 2010; Ferguson et al., 2017). Santiesteban et al. (2014) demonstrated that when the avatar was replaced by a non-social entity, say, an arrow, consistent results emerge. This leads to questions as to whether consistency is influenced by mentalizing, or whether this is a domain-general process that does not involve mentalizing. For example, the central stimulus in the present study (i.e., the avatar’s nose or eyes) merely acted as a directional cue for orienting attention, which adds a lag in RTs during inconsistent trials (Catmur et al., 2016). Conversely, some studies manipulated the avatar’s head position or line of sight access, and the results of dissociation in different conditions favored an implicit mentalizing account (Baker et al., 2016; Gardner M. et al., 2018). We observed different response patterns when participants performed the dot perspective task. In both Experiments 1 and 2, if the avatar was only a directional cue, there should not have been a significant difference response patterns between the chess play and the non-chess player. Therefore, our findings provide further evidence against the suggestion that the avatar task is purely driven by the spatial cueing of attention (e.g., Heyes, 2014; Santiesteban et al., 2014). Here, the avatar is more than just a directional cue, and subsequent perspective shifting was required (Baker et al., 2016; Michael et al., 2017; Gardner M.R. et al., 2018).

From a developmental perspective, the finding provided insight into the attenuation of egocentric biases through development. Specifically, social experiences accumulated over the years leading to adulthood may help adults inhibit their own perspective when assessing others’. It is possible that as experts in social domain, adults rely on an entirely different psychological process for perspective taking (Epley et al., 2004).

There were nevertheless some caveats in this study. Firstly, a cross-sectional design by nature, the findings of the current study do not indicate causality. Historical data are not available to compare the chess players and the non-players when they were 6-year-old before the chess group started their formal training. Although the two groups scored the same on both academic performances and teacher’s ratings on the subjects of Chinese and mathematics at the time of the current study, it is still possible that there were differences in domain-general cognitive abilities between the two groups. Experimental design and training studies are warranted to identify the causal relations between chess experience and perspective taking ability. In addition, the sample size of the current study is small, due to the highly specialized chess-playing participant pool. Furthermore, data regarding the actual level of the chess players’ skill are not available. It is possible that the chess players’ strength might be a valuable predictor of their perspective taking ability. Longitudinal and training studies with larger samples and more rigorous controls in the future are warranted to establish causal association between chess training and visual perspective training ability.

Secondly, the result of the current study could not rule out the possibility that EF mediates the effect of chess training on perspective taking. The association between chess training and EF is well established (Nichelli et al., 1994; Atherton et al., 2003; Unterrainer et al., 2006; Demily et al., 2009; Goncalves et al., 2014), so is the association between EF and perspective taking during childhood and beyond (Devine and Hughes, 2014; Wang et al., 2016). It is possible that long term chess training improves children’s domain general cognitive capacity such as cognitive flexibility and working memory, which in turn enhances their perspective taking. Future research should measure players’ EF and explore its mediation effect in the association between chess experience and perspective taking.

More importantly, it remains an empirical question whether the visual perspective taking paradigm reflects social perspective taking in real life social exchange. The empirical evidence is currently mixed on whether visual perspective taking tasks are truly mentalizing tasks, especially the Level-I visual perspective taking tasks (Pearson et al., 2013; Conway et al., 2017). The Level-II visual perspective taking tasks adopted in the current study, on the other hand, do arguably involve the cognitive mechanism of standing in other people’s shoes (Pearson et al., 2013), which is supported by our result. In real life chess games, however, social cues are involved in players’ decision making. Future studies of the cognitive benefit of chess training should consider more relevant and ecologically valid perspective taking tasks.

## Ethics Statement

This study was carried out in accordance with the recommendations of Guidelines for Research with Human Subjects, the Research Ethics Committee of the Shaoxing University on Activities Involving Human Subjects with written informed consent from all subjects. The protocol was approved by the Research Ethics Committee of the Shaoxing University and the participating schools. Informed consent was obtained from parents to allow their child to participate in the study.

## Author Contributions

QG, WC, ZW, and DL contributed to the conception and design of the study. QG, WC, and ZW performed the statistical analysis and wrote the first draft of the manuscript. DL wrote the sections of the manuscript. All authors contributed to the manuscript revision, and read and approved the submitted version of the manuscript.

## Conflict of Interest

The authors declare that the research was conducted in the absence of any commercial or financial relationships that could be construed as a potential conflict of interest.
